# *Period*-independent novel circadian oscillators revealed by timed exercise and palatable meals

**DOI:** 10.1038/srep21945

**Published:** 2016-02-24

**Authors:** Danilo E. F. L. Flôres, Crystal N. Bettilyon, Shin Yamazaki

**Affiliations:** 1 Department of Neuroscience, University of Texas Southwestern Medical Center, 5323 Harry Hines Blvd., Dallas, TX, 75390-9111 USA; 2 Institute of Biosciences, University of São Paulo, Rua do Matao – Travessa 14, 321, São Paulo, SP, 05508-900, Brazil

## Abstract

The mammalian circadian system is a hierarchical network of oscillators organized to optimally coordinate behavior and physiology with daily environmental cycles. The suprachiasmatic nucleus (SCN) of the hypothalamus is at the top of this hierarchy, synchronizing to the environmental light-dark cycle, and coordinates the phases of peripheral clocks. The *Period* genes are critical components of the molecular timekeeping mechanism of these clocks. Circadian clocks are disabled in *Period1/2/3* triple mutant mice, resulting in arrhythmic behavior in constant conditions. We uncovered rhythmic behavior in this mutant by simply exposing the mice to timed access to a palatable meal or running wheel. The emergent circadian behavior rhythms free-ran for many cycles under constant conditions without cyclic environmental cues. Together, these data demonstrate that the palatable meal-inducible circadian oscillator (PICO) and wheel-inducible circadian oscillator (WICO) are generated by non-canonical circadian clocks. Entrainment of these novel oscillators by palatable snacks and timed exercise could become novel therapeutics for human conditions caused by disruptions of the circadian clocks.

Anticipation of daily changes in the environment is believed to improve the fitness of living organisms^1^. This anticipation is conferred by circadian clocks that control ~24-h rhythms of physiology and behavior. The mammalian circadian system has a hierarchical chrono-architecture^2^,^3^. At the top of this hierarchy is the master circadian pacemaker, located in the suprachiasmatic nucleus (SCN) of the brain. The SCN entrains to the light-dark cycle, which is often the dominant environmental factor, and orchestrates the ensemble of peripheral clocks. These peripheral clocks then regulate local physiological processes.

The molecular circadian machinery has been extensively studied^4^. In mammals, a self-sustaining, cell-autonomous circadian rhythm is generated by interconnected molecular feedback loops of gene transcription and translation^5^. CLOCK and BMAL1 are positive regulators that activate transcription of *Period (Per)*, *Cryptochrome (Cry)* and other genes. PERIOD and CRYPTOCHROME, in turn, form a complex with other proteins and negatively regulate their own transcription^6^. This molecular circadian feedback loop is universal among all known clocks in the brain and peripheral tissues^7^.

Although the light-dark cycle is often the dominant environmental signal, food availability is also critical for the survival of organisms^8^,^9^. Animals anticipate the timing of food availability via the food-entrainable oscillator (FEO). In rodents, locomotor activity increases several hours before food availability (food anticipatory activity, FAA) when a single meal is provided daily at a fixed time (restricted feeding)^10^. It is also well demonstrated that restricted feeding entrains clocks in peripheral tissues^11^,^12^,^13^; however, a recent study suggests that this may be a mechanism independent from the appearance of FAA^14^. This food anticipatory activity (the output of the FEO) observed during restricted feeding immediately disappears when the animal is released to *ad libitum* feeding, but re-appears when the animal is subsequently food-deprived. The persistence of FAA during fasting demonstrates the endogenous (self-sustaining) characteristic of the FEO. However, because mice can only be fasted for ~48 h, the free-running rhythm of the FEO cannot be observed. Notably, food-anticipatory activity appears even when the SCN is lesioned, but the anatomical location of the FEO remains unknown (despite exhaustive searches)^15^. The identification of the FEO would be facilitated if the free-running rhythm of the FEO could be observed.

Rodents anticipate rewarding stimuli, including scheduled access to water, stimulants, and palatable meals^16^,^17^,^18^,^19^,^20^,^21^,^22^,^23^. In the current study, we sought to develop an approach to expose the free-running rhythm of the FEO by providing scheduled access to a high-fat/high-sugar palatable meal in the presence of *ad libitum* chow. Instead, using this approach we discovered a novel non-canonical circadian clock, the palatable meal-inducible circadian oscillator (PICO). Similarly, we found that entrainment to timed access to a running wheel was dependent on a non-canonical circadian clock, the wheel-inducible circadian oscillator (WICO). Entrainment of these novel oscillators by palatable snacks and timed exercise could become novel therapeutics for human conditions caused by disruptions of the circadian clocks.

## Results

### Wild-type mice anticipate palatable meals

We first used a training protocol that combined restricted feeding of both chow and a palatable meal (peanut butter) to establish palatable meal anticipatory activity (PAA) in wild-type mice in the light-dark cycle (see Supplementary Fig. S1; the protocol was slightly modified from Keith *et al.*^22^). After training, mice were provided with *ad libitum* chow and given peanut butter (for 1 h) at either ZT4 or ZT10 (Supplementary Fig. S2). Consistent with Keith *et al.*^22^, the robustness of PAA was phase-dependent; all mice fed peanut butter at ZT4 (6 of 6) exhibited PAA, but only 4 out of 7 mice displayed PAA when fed at ZT10. We next tested whether PAA persisted in constant conditions by removing the daily palatable meal. After termination of daily palatable meal access, mice in both the ZT4 (5 of 6) and ZT10 (3 of 7) groups had sustained PAA when provided only with *ad libitum* chow (Supplementary Figs S2 and S3). These data suggest that there is an endogenous, self-sustained oscillator entrained by palatable food. There was no correlation between the amount of peanut butter consumed and robustness of anticipatory activity (Supplementary Fig. S4).

**Figure 1:**
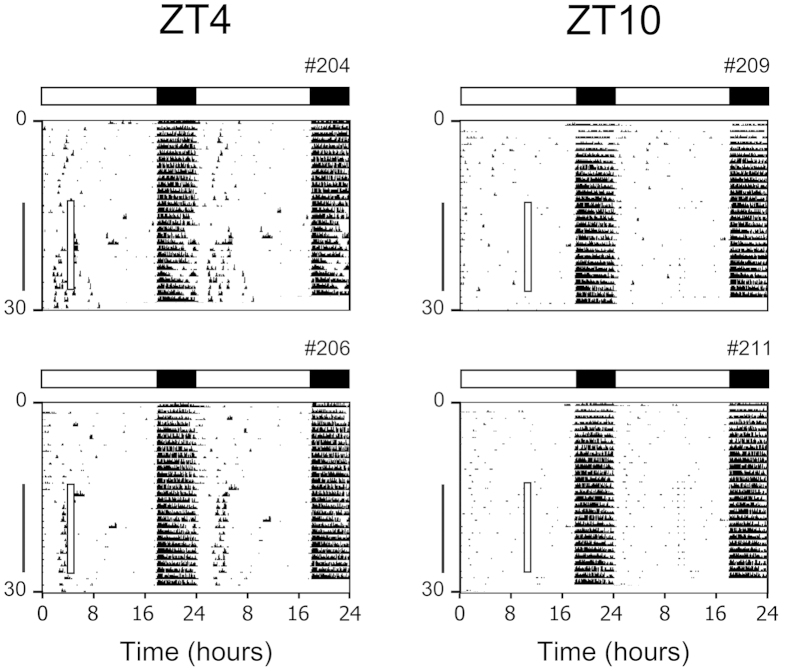
Wild-type mice display anticipatory activity to a daily palatable meal. Representative double-plotted actograms of wheel-running activity of wild-type male C57BL/6J mice singly housed in 18L:6D (indicated by white and black bars) with *ad libitum* chow for the entire experiment. From days 14 to 27 (indicated by vertical line), peanut butter was placed in the cage for 1 h (indicated by the open square box on the left of each actogram) at either ZT4 (4 h after lights-on) or ZT10 (10 h after lights-on). Mice were then maintained in *ad libitum* feeding conditions for 3 days (days 28–30; no peanut butter feeding). Individual actograms from all mice are shown in Supplementary Fig. S5.

It is possible that the PAA we observed resulted from entrainment of the FEO by the scheduled restricted feeding of chow during the training portion of the protocol. To address this potential caveat, we asked whether mice would entrain to a daily palatable meal without the training step (i.e. with no chow restricted feeding). For this, mice were maintained with *ad libitum* chow and peanut butter was provided for 1 h at ZT4 or ZT10. For ZT4, we found that 4 out of 6 mice expressed PAA starting 1–2 h before peanut butter was placed in the cage (Fig. 1 and Supplementary Fig. S5). PAA persisted for 2 days after termination of daily peanut butter feeding (Supplementary Fig. S6). In contrast, none of the mice (0 of 6) fed peanut butter at ZT10 developed PAA. There was no correlation between the amount of peanut butter consumed and robustness of anticipatory activity (Supplementary Fig. S7). These data demonstrate that mice developed PAA to scheduled peanut butter during *ad libitum* chow and PAA persisted when the palatable meal was removed. Moreover, training the mice with coincident restricted chow and scheduled peanut butter was not necessary for the development of palatable meal anticipatory activity.

### The palatable meal-inducible circadian oscillator is a novel non-canonical oscillator

**Figure 2:**
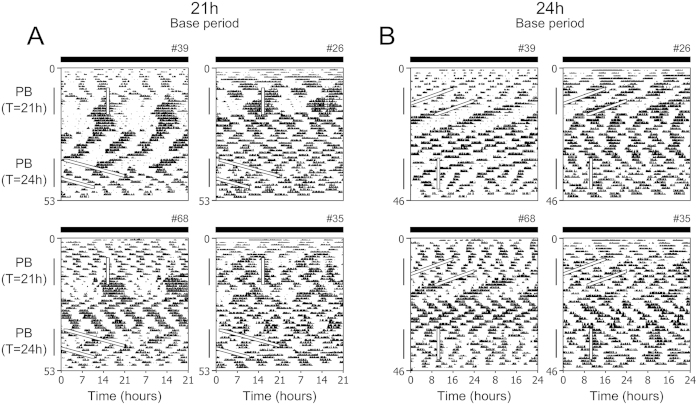
The PICO in *Per1/2/3* triple mutant mice has a 21-h period that persists in constant conditions. Representative double-plotted actograms of wheel-running activity of *Per1/2/3* triple mutant mice kept in constant darkness (indicated by black bars) with *ad libitum* chow throughout the experiment. Actograms are plotted with either a 21-h period (**A**) or 24-h period (**B**). Mice were fed peanut butter for 1 h each cycle on a 21-h cycle (PB: T = 21 h) and then released into constant conditions (no peanut butter). Then mice were given peanut butter for 1 h each cycle on a 24-h cycle (PB: T = 24 h) and then released into constant conditions. All individual actograms are shown in Supplementary Fig. S8. Male: #39 and 68. Female: #26 and 35.

In our initial experiment, we could not rule out the possibility that PAA was driven by the SCN. Thus, we next tested whether timed palatable meal could reveal PICO in arrhythmic *Period* mutant mice, in which the molecular clocks in the SCN and peripheral organs are disabled. In constant conditions (*ad libitum* chow and constant darkness) *Per1/2/3* triple mutant mice did not have circadian rhythms of wheel-running activity, demonstrating that the SCN was disabled in these mice (Fig. 2, Supplementary Fig. S8; they displayed ultradian behavior rhythms typical of circadian mutant mice). We gave periodic 1-h peanut butter access to *Per1/2/3* triple mutant mice in constant darkness with *ad libitum* chow (Fig. 2, Supplementary Fig. S8). When peanut butter was given at a 21-h interval (T21), half of the mice (4 of 8) showed consolidated PAA with a 21-h period (Fig. 2, Supplementary Fig. S8; mice #39, 26, 68 and 02). Another mouse developed a ~17-h period PAA rhythm (Supplementary Fig. S8; #27). The remaining 3 mice did not develop consolidated activity prior to peanut butter feeding (Fig. 2 and Supplementary Fig. S8; #29, 30 and 35); however, the ultradian rhythmicity in these mice was affected by the treatment. There was no correlation between the development of consolidated activity and peanut butter consumption (Supplementary Fig. S9).

Surprisingly, the palatable meal-induced rhythm continued for up to 7 cycles after the termination of peanut butter feeding. The free-running periods of these rhythms were ~21-h (Supplementary Table S1). We then fed the mice peanut butter for 1 h each day on a 24-h cycle (T24). None of the *Per1/2/3* triple mutant mice developed PAA to the 24-h palatable meal schedule (Fig. 2, Supplementary Figs S8 and S9). In addition, the T24 peanut butter feeding did not alter the ultradian activity rhythm. Thus, the PAA rhythm has two fundamental properties of a circadian oscillator: it has a range of entrainment (e.g. entrains to T21 but not T24) and it persists in constant conditions. Together, these data suggest that the PAA rhythm is generated by a circadian oscillator. We named this novel circadian oscillator the palatable meal-inducible circadian oscillator (PICO). Moreover, because PICO persists in the absence of functional PERIOD 1, 2, and 3, it is a non-canonical circadian clock.

### The period of the methamphetamine sensitive circadian oscillator is equivalent to PICO

**Figure 3:**
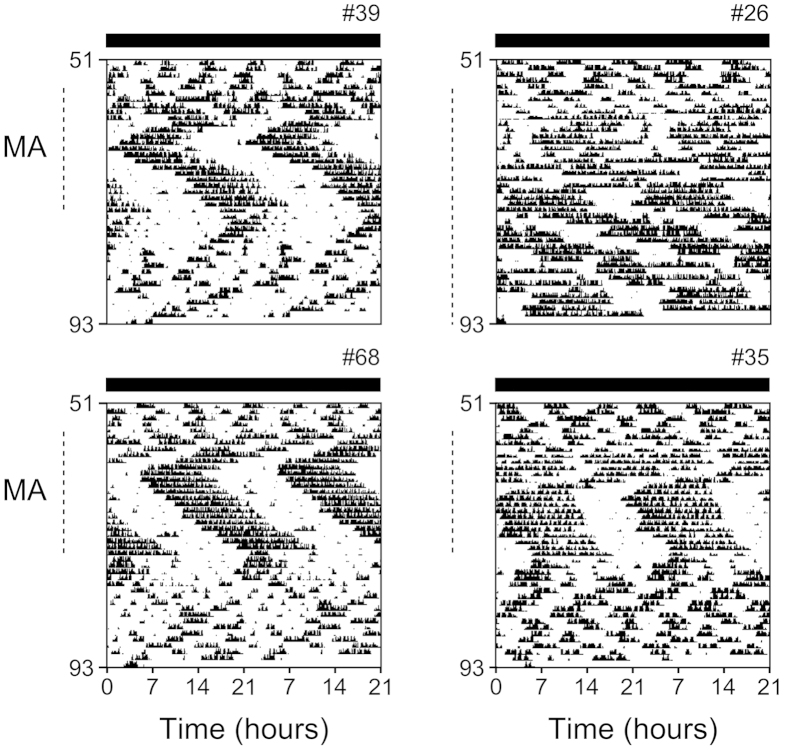
MASCO driven behavior rhythms in *Per1/2/3* triple mutant mice. Representative double-plotted actograms (the same mice shown in Fig. 2) of wheel-running activity of *Per1/2/3* triple mutant mice given methamphetamine (MA; indicated by dotted lines to the left of the actograms) in their drinking water (0.005%) in constant darkness. Actograms are plotted with a 21-h period. All individual actograms are shown in Supplementary Fig. S10.

The methamphetamine sensitive circadian oscillator (MASCO or methamphetamine-induced oscillator, MAO) is a non-SCN circadian oscillator. The rhythmic output of MASCO can only be observed when low dose methamphetamine is chronically administered^24^,^25^,^26^,^27^. We measured the MASCO period from the same mutant mice used in the timed palatable meal experiments. After the palatable meal-induced behavior rhythms (PICO outputs) were extinguished (evidenced by the presence of ultradian, but no circadian rhythms), mice were administered low-dose methamphetamine (0.005%) in their drinking water. Consistent with our previous study, all *Per1/2/3* mutant mice exhibited consolidated activity^28^. Mice initially had a ~18-h period of wheel-running activity rhythm that persisted for several days and then switched to a stable 22-h period (Fig. 3, Supplementary Fig. S10 and Table S1).

### Timed access to a running wheel reveals the wheel-induced circadian oscillator

**Figure 4:**
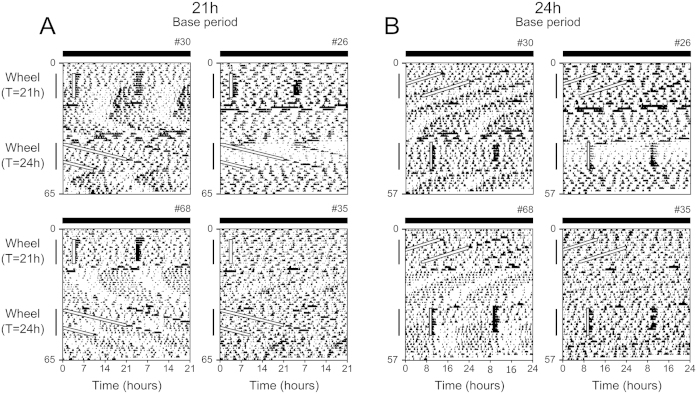
Timed voluntary wheel activity reveals an endogenous circadian activity rhythm in *Per1/2/3* triple mutant mice. Representative double-plotted actograms of general activity of *Per1/2/3* triple mutant mice recorded by passive infrared motion detectors in constant darkness with *ad libitum* chow and a locked running wheel. Actograms are plotted with either a 21-h period (**A**) or 24-h period (**B**). The wheel was unlocked for 1 h each cycle on a 21-h interval (Wheel: T = 21 h) and then released into constant conditions (continuously locked wheel). Then the wheel was unlocked for 1 h each cycle on a 24-h cycle (Wheel: T = 24 h) and then released into constant conditions. All individual actograms of general activity and wheel revolutions are shown in Supplementary Fig. S11. Male: #30 and 68. Female: #26 and 35.

Mice develop anticipatory activity to scheduled palatable meals, and low doses of methamphetamine reveal rhythmic behavior. Both of these stimuli are potent rewarding stimuli that activate the dopaminergic system^18^,^20^,^29^,^30^. Likewise, voluntary running wheel activity elevates dopamine in the rodent brain^31^,^32^. Therefore, we tested if timed access to a running wheel would also elicit a circadian rhythm in *Per1/2/3* mutant mice. Six of the *Per1/2/3* triple mutant mice used in the palatable meal experiments were kept in constant darkness and housed with a locked running wheel. The wheel was unlocked for 1 h each cycle on a 21-h interval (T21). When the wheel was unlocked, 5 of the 6 mice ran on the wheels (Fig. 4, Supplementary Figs S11 and S12). Unlike the PAA during scheduled peanut butter access, mice did not anticipate the recurrent unlocking of the wheel. Instead, general activity, measured by passive infrared sensors, increased shortly after the wheel was unlocked and remained elevated for 1–2 h after the wheel was relocked. The consolidated wheel-induced activity developed in 3 of the 6 mice (Fig. 4 and Supplementary Fig. S11; mice #68, 29 and 30). The 3 mice that exhibited rhythmic behavior had the most wheel-running revolutions during the 1 h of wheel access (Supplementary Fig. S12).

When the mice were released into constant conditions (the wheel remained locked), the wheel-induced rhythm free-ran for many days with a ~21-h period (Supplementary Table S1). Interestingly, in one of the cages the wheel-locking mechanism failed during constant conditions and the wheel was unlocked for a day. The consolidated 21-h activity rhythm of the mouse in this cage quickly changed to an ultradian rhythm (Supplementary Fig. S11; #29).

We then gave the same *Per1/2/3* mutant mice 1 h of wheel access on a 24-h interval (T24). Two (out of 6) mice developed consolidated activity. When the wheel was permanently locked, their rhythms free-ran with periods of 21-h and 23-h, respectively. Throughout the course of our experiments, only one mouse (#68) exhibited consolidated activity in all three treatments (peanut butter, methamphetamine, running wheel). With the exception of this mouse, the mice that displayed PAA (#39, 26, 27, 02) did not display wheel-induced consolidated rhythms. Similarly, the mice that showed wheel-induced rhythms (#29, 30) did not display PAA.

## Discussion

In this study we discovered 2 stimuli, scheduled palatable meals and timed voluntary exercise, that reveal free-running circadian rhythms that do not rely on the canonical molecular timekeeping mechanism. Because scheduled palatable meals and wheel access induced circadian behavior rhythms that persisted in constant conditions, we believe these rhythms are the output of non-canonical circadian oscillators, PICO and WICO, respectively.

It is unclear whether PAA and wheel-induced activity are controlled by a single circadian oscillator (i.e. PICO and WICO are the same oscillator). However, it should be noted that 2 different behavioral outputs were measured – wheel-running activity for PICO and general activity for WICO. This could account for differences in the phases of the induced rhythms.

It is tempting to speculate that either PICO or WICO (or both) are the same circadian oscillator as the FEO and MASCO. Indeed, PICO and WICO share characteristics with the FEO and MASCO. First, all of these oscillators require treatment in order to express their behavioral outputs (unlike the SCN that controls numerous behavior rhythms without requiring a stimulus). Second, all of these oscillators are present in *Per1/2/3* triple mutant mice, demonstrating that they use a non-canonical molecular timekeeping mechanism. Finally, the periods of the output rhythms of all four oscillators are ~21-h in *Per1/2/3* triple mutant mice.

Despite these similarities, PICO and WICO also express characteristics that are distinct from the FEO and MASCO. Unlike the FEO, both PICO and WICO have persistent self-sustained rhythms that continue in the absence of cyclic signals. Unlike MASCO, WICO requires timed wheel access to reveal its rhythm, as evidenced by the finding that *Per1/2/3* mutant mice with *ad libitum* wheel access do not express a ~21-h behavior rhythm. Likewise, PICO differs from MASCO because the output of PICO is observed only when the rhythmic input is close to the endogenous PICO period. This cyclic external input is not necessary to reveal MASCO (constant infusion of methamphetamine can reveal MASCO^25^). We chose a standard procedure to reveal MASCO (methamphetamine was administered in the drinking water). However, it has been shown that mice exhibit anticipatory activity to daily injections of methamphetamine^18^. It will be interesting to see if T21 methamphetamine injection reveals a PICO/WICO like behavior rhythm.

The mysteries of these reward-related oscillators can only be solved by discovering the anatomical loci of these clocks. To this end, we have established 2 new experimental tools for identifying the underlying neural substrates of PICO and WICO (and perhaps the FEO and MASCO). Several studies have shown that it is difficult to produce even weak anticipatory activity to scheduled palatable meals in mice^21^,^33^. In contrast, Keith *et al.* reported robust anticipatory activity to a daily offering of peanut butter in mice using a training protocol that combined scheduled chow and palatable meal training^22^. In the current study we established a new simplified protocol (that does not require training) to establish PAA in mice during *ad libitum* chow access. Moreover, using this method, we can measure the free-running period of PAA in wild-type and circadian mutant mice.

Scheduled access to a wheel also induces robust wheel-induced activity. To perform these experiments, we designed an automated wheel lock-and-release system that is controlled by the computer. Unlike restricted feeding of chow to entrain the FEO or scheduled feeding of palatable meals to entrain PICO, this new automated method requires very little investigator effort to reveal a robust wheel-induced rhythm that free-runs when the wheel is permanently locked.

Beyond the relevance to the basic understanding of the mammalian circadian system and regulation of complex behavior, our findings also have translational impact. In this study we showed that timed exercise and palatable snack (at the proper timing) are potent entraining stimuli. Thus, these highly accessible methods may be used to treat human disorders caused by disruptions of circadian rhythms.

## Methods

### Animals

Wild-type male C57BL/6J mice were obtained from the E. K. Wakeland Mouse Breeding Core (UT Southwestern, Dallas, TX USA) or from our breeding colony at UT Southwestern. *Per1/2/3* triple mutant mice were generated by intercrossing *Per1*^+/−^*/Per2*^−/−^*/Per3*^−/−^ mice (C57BL/6J N15) for 4 to 6 generations in our breeding colony^28^. All mice were bred and maintained in 12L:12D illuminated by fluorescent bulbs (Ecolux, 32W). Weaned mice were group-housed in cages without running wheels with *ad libitum* chow and water (20–23 °C and 18–68% relative humidity). Male wild-type C57BL/6J (7–18 weeks old) and male and female *Per1/2/3* triple mutant mice (10–55 weeks old) were used for experiments. All experiments were conducted in accordance with the guidelines of the National Institutes of Health Guide for the Care and Use of Laboratory Animals. All procedures were approved by the Institutional Animal Care and Use Committee at UT Southwestern Medical Center (Protocol#: 2013-0035).

### Activity recordings

Circadian behavior recordings were conducted in light-tight ventilated boxes (22–23 °C, 19–54% relative humidity). Light (7 μW/cm^2^/s, 55 lux inside the cage) was generated by green LEDs controlled by Chamber Controller (Phenome Technologies, Inc Chicago, IL USA). Mice were singly housed in plastic cages (length × width x height: 29.5 × 11.5 × 12.0 cm) with running wheels (diameter 11 cm). Wheel revolutions were continuously recorded every minute by the ClockLab system (Actimetrics, Wilmette, IL USA). A passive infrared sensor (product ID 189, Adafruit, NYC, NY USA) located 10 cm above the cage lid was used to monitor general activity in the timed wheel access experiment. Cages and water bottles were changed once every 3 weeks. An infrared viewer (FIND-R-SCOPE Infrared Viewer; FJW Optical Systems, Inc. Palatine, IL USA) was used to perform maintenance and feeding in the dark without exposing mice to visible light.

### Restricted feeding and timed palatable meal feeding

Restricted feeding of chow was done manually by placing and removing chow (Teklad Global 18% Protein Rodent Diet 2918; Harlan, Madison WI USA) on the bottom of the cage. Peanut butter (Jif® Creamy Peanut Butter, 50% fat, 25% carbs and 22% protein; The J.M. Smucker Company, Orrville, OH USA) was added and removed manually in a 35 mm plastic petri-dish lid on the bottom of cage. The protocols are detailed in Supplementary Fig. S1 and the time of each treatment is indicated on each actogram.

### Methamphetamine

During methamphetamine treatment, a bottle containing regular water was replaced with a bottle containing 0.005% methamphetamine (Sigma-Aldrich, Inc. St. Louis, MO USA). The time when methamphetamine water was available is indicated on each actogram.

### Timed access to running wheel

A large push-pull solenoid (product ID 413, Adafruit, NYC, NY USA) was placed on the cage lid and blocked the rotation of the running wheel. The wheel was unlocked or locked via the Pick and Hold Solenoid Driver Module (PH-ET-01, Optimal Engineering Systems, Inc, Van Nuys, CA, USA) controlled by ClockLab. The pickup voltage (24 V) was set for 1.2 sec and followed by a hold voltage (3 V). The schedule for wheel-unlocking is indicted on each actogram.

### Data analysis

Each actogram was generated using 6-min bins and the percentile option in the ClockLab analysis software. Twenty-four hour group average activity profiles were generated from 6-min bin activity files using ClockLab. For each activity profile, either 3 days of *ad libitum* chow, all days during 1-h peanut butter access, or the first 3 days of *ad libitum* chow after peanut butter treatment were averaged for individual mice. Then group average profiles were generated by averaging individual activity profiles. PAA was defined as the total number of wheel revolutions during the 2 h prior to peanut butter access. The individual daily average of anticipatory activity during peanut butter feeding was used for PAA analysis. Mean peanut butter consumption was compared between groups by the Wilcoxon-Mann-Whitney unpaired two-tailed test. Spearman’s correlation test was used for correlation analysis. For both statistical tests, alpha level was set to 0.05. The free-running period was calculated by fitting a regression line to the onset of activity detected by ClockLab (default criteria settings). The days used for period analysis (at least 5 cycles) are indicated on each individual actogram in the supplemental figures.

## Additional Information

**How to cite this article**: Flôres, D. E. F. L. *et al.* *Period* - independent novel circadian oscillators revealed by timed exercise and palatable meals. *Sci. Rep.* **6**, 21945; doi: 10.1038/srep21945 (2016).

## Supplementary Material

Supplementary Information
