# Supplementary material for: Period-independent novel circadian oscillators revealed by timed exercise and palatable meals
Source: Sci Rep. 2016 Feb 24;6:21945. doi: 10.1038/srep21945 (PMC4764932; doi:10.1038/srep21945)
Supplement: Supplementary Information [file srep21945-s1.pdf]

[Supplementary Information]

Period-independent novel circadian oscillators revealed by timed exercise and palatable meals

Danilo E. F. L. Flôres, Crystal N. Bettilyon and Shin Yamazaki

Table S1. Periods of emergent rhythms in *Per1/2/3* triple mutant mice under different conditions.

	Period of consolidated rhythm (h)			
Mouse ID	T21 peanut butter	Methamphetamine	T21 wheel	T24 wheel
#68 (male)	21.16	18.86 | 22.29**	21.71	23.45
#29 (male)	UR	22.69	20.86	UR
#30 (male)	UR	21.19 | 19.55 | 21.34	20.69 | 22.86	20.96
#39 (male)	20.78	18.95 | 22.62**	NT	NT
#26 (female)	19.81	25.27	UR	UR
#27 (female)	17.19	19.24	NT	NT
#02 (female)	21.05*	20.51 | 24.77 | 18.37	UR	UR
#35 (female)	UR	17.61 | 21.88**	UR	UR

NT: Not tested (mouse was removed from the study). UR: Ultradian rhythm (no consolidated circadian rhythm). Days used for period determination are indicated by brackets on the right side of the actograms in Figure S8, 10, 11. *: Bimodal rhythm. **: Stable rhythm induced by methamphetamine.

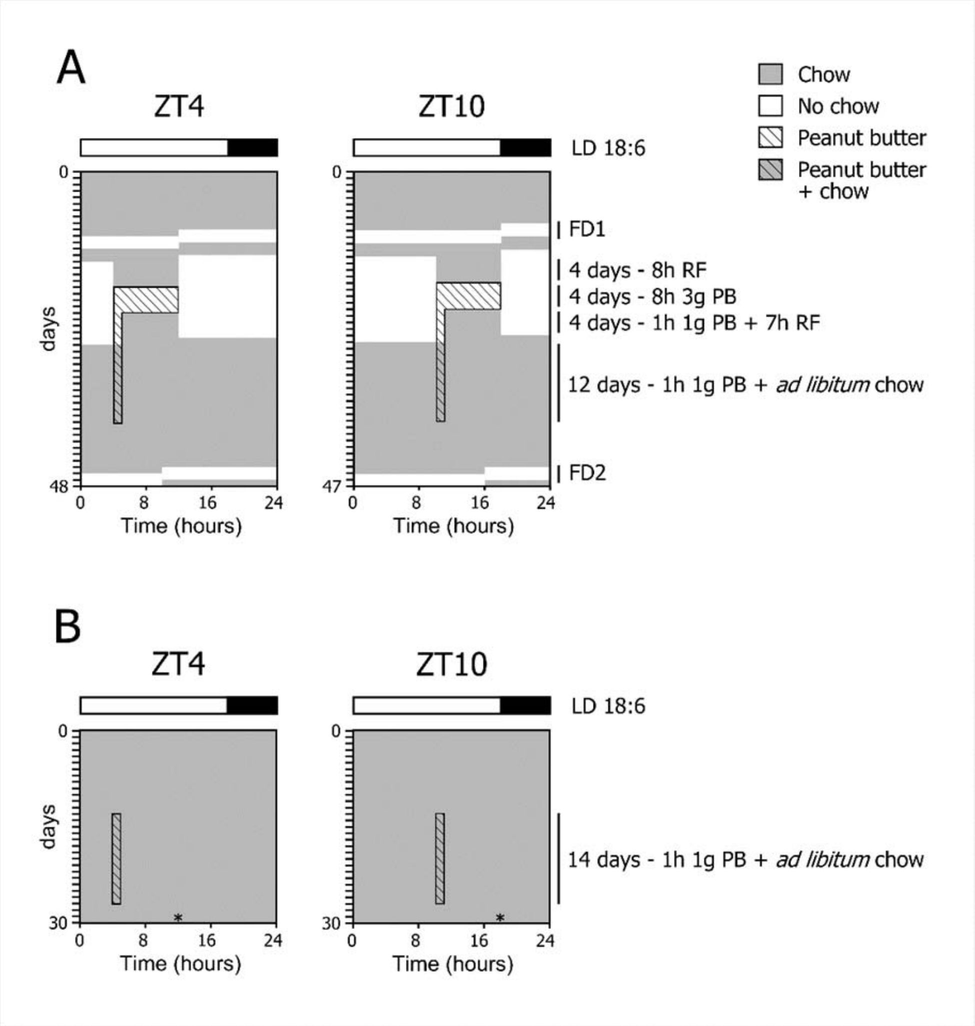

Figure S1. Protocols for observing palatable meal anticipatory activity. Two protocols with (A) or without (B) training steps are shown. Gray areas represent the times of regular chow availability. White areas indicate the absence of chow. The time when peanut butter was given is depicted by the areas filled with diagonal lines. The experimental conditions are indicated on the right side of each panel. RF: restricted feeding. PB: peanut butter treatment. FD: food deprivation. The white and black bar on top of each panel indicates the light-dark cycle. *: light and dark cycle was shifted for another experiment.

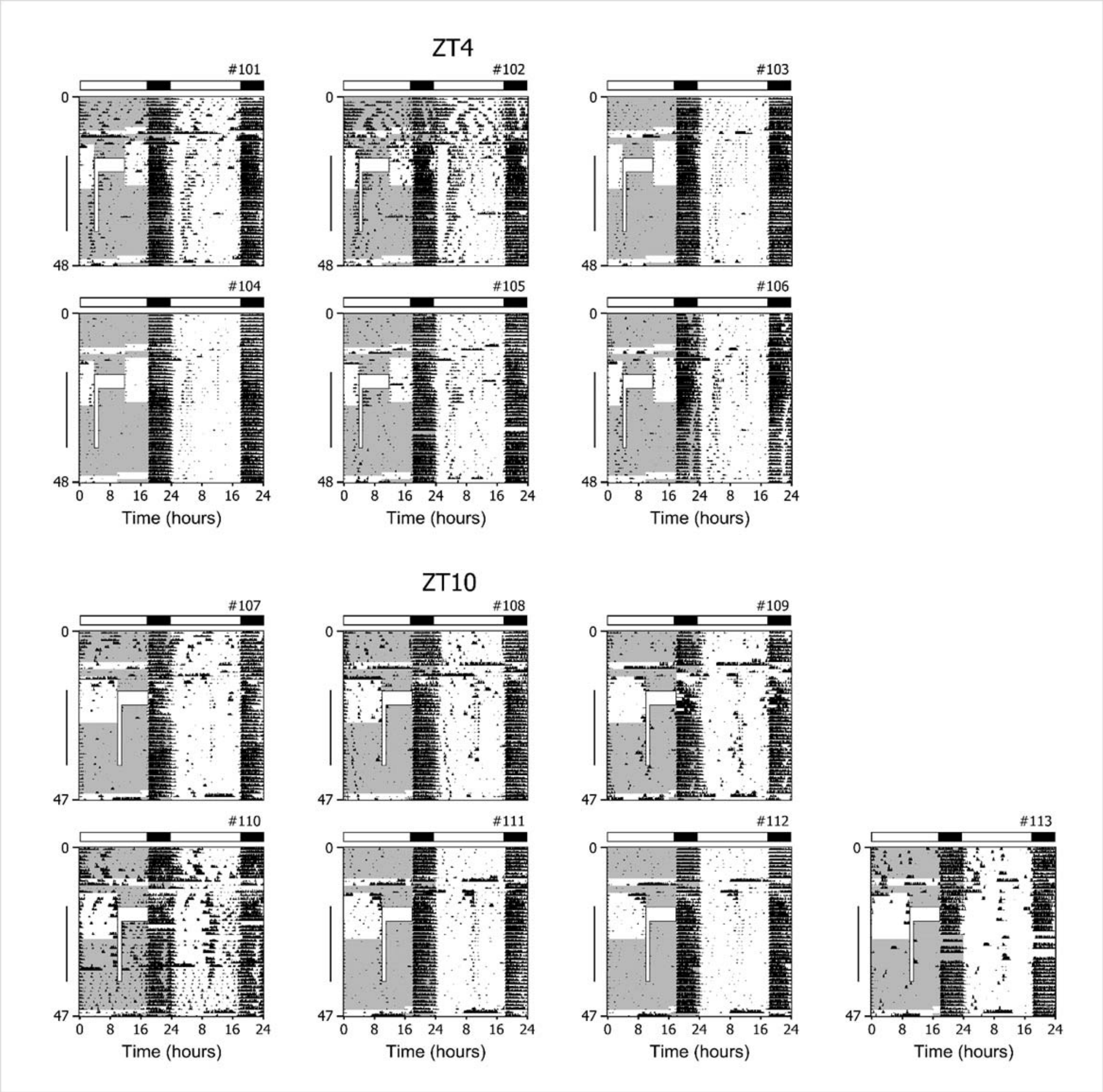

Figure S2. Wild-type mice display anticipatory activity to a daily palatable meal.
Double-plotted actograms of wheel-running activity of wild-type male C57BL/6J mice singly housed in 18L:6D (indicated by white and black bars). Scheduled restricted feeding of chow was paired with scheduled palatable meal access (days 15-27) to develop palatable meal anticipatory activity (training protocol in Figure S1A). From days 28-39, mice received only daily palatable meals with *ad libitum* chow. On the left panel of each actogram, the time when chow was available is indicated by gray shading and the time when peanut butter was available is indicated by a white box. Peanut butter was given at either ZT4 (top panel) or ZT10 (bottom panel). A vertical line to the left of each actogram shows days of peanut butter presentation.

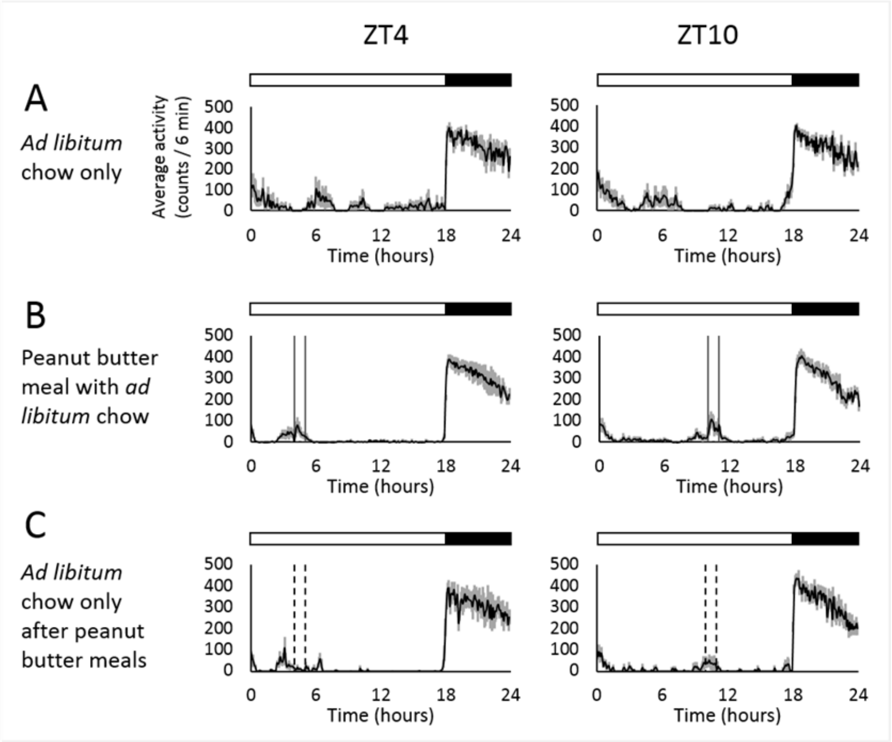

Figure S3. Group average activity profiles of data presented in Figure S2. Group average activity profiles (6-min bins) showing; A: the last 3 days of *ad libitum* chow, B: all days of 1-h peanut butter access (indicated by two vertical lines), and C: the first 3 days after termination of peanut butter feeding (previous peanut butter time indicated by dotted lines). Peanut butter was given at ZT4 (left column) or ZT10 (right column). White and black bars above each graph represent the light-dark cycle.

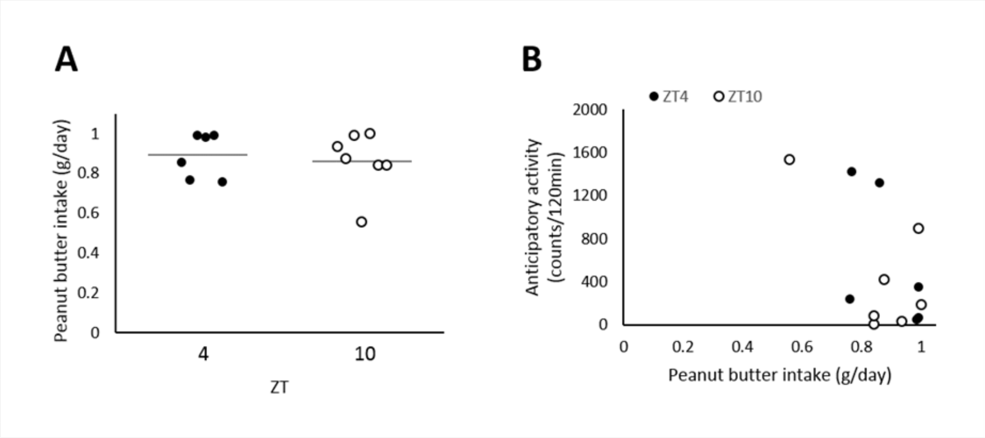

Figure S4. Peanut butter consumption is not correlated with the robustness of palatable meal anticipatory activity (from the experiment in Figure S2). Peanut butter consumption was calculated as an average of daily peanut butter intake for the last 12 days of peanut butter access. Anticipatory activity was calculated as the total number of wheel revolutions during the 2 h before peanut butter access (average of the last 12 days of peanut butter access). A: There was no difference in peanut butter consumption between mice fed peanut butter at ZT4 vs. ZT10 ($p=0.94$ Mann-Whitney). Horizontal lines indicate the group average. B: There was no correlation between robustness of palatable meal anticipatory activity and peanut butter intake ($p=0.31$ Spearman's correlation coefficient).

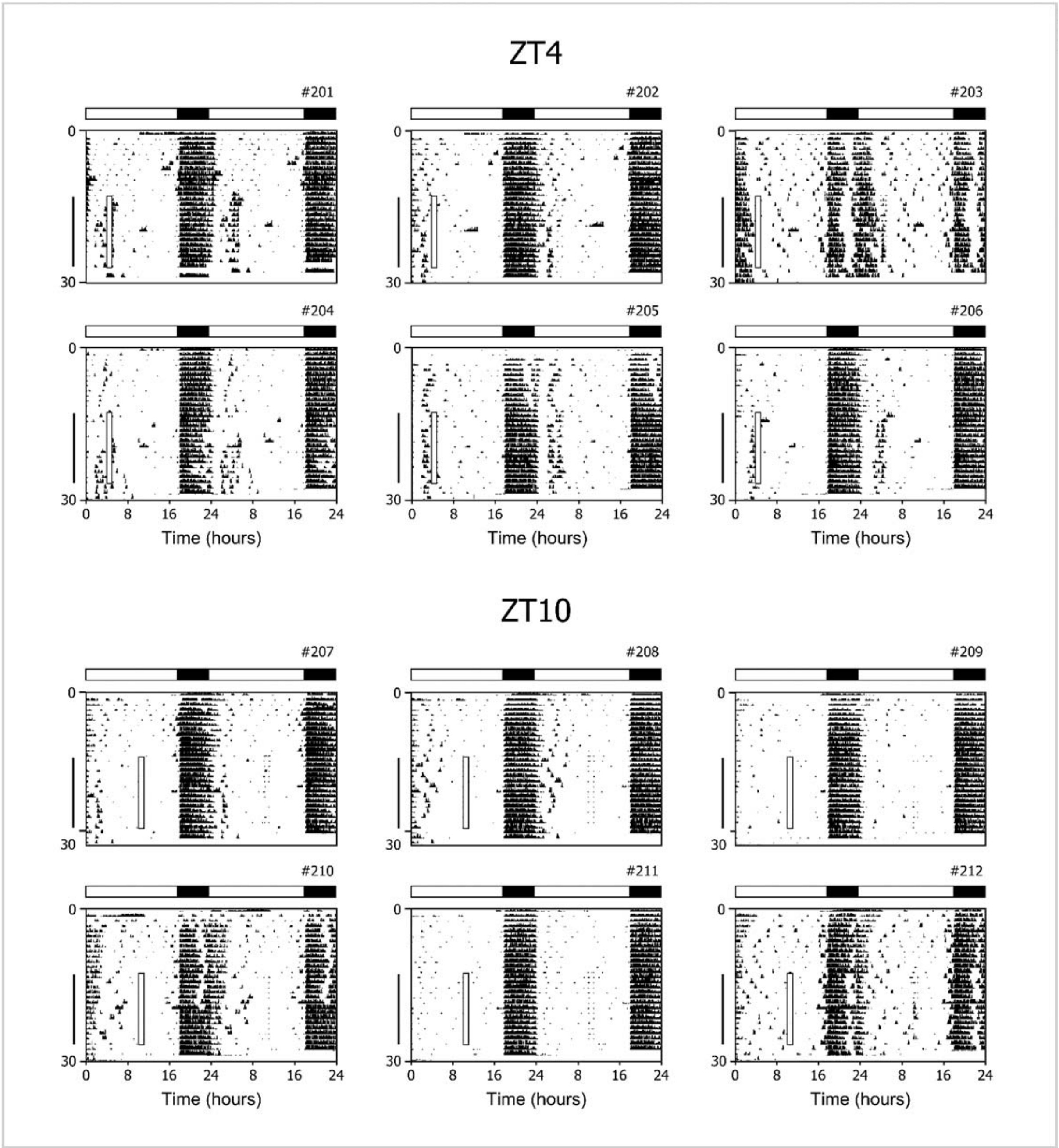

Figure S5. Palatable food anticipatory activity in naïve wild-type mice without food restriction. Double-plotted actograms of wheel-running activity of wild-type male C57BL/6J mice singly housed in 18L:6D (indicated by white and black bars) with *ad libitum* chow for the entire experiment. From days 14 to 27 (indicated by vertical line), peanut butter was placed in the cage for 1 h (indicated by the open square box on the left side of each actogram) at either ZT4 or ZT10 (protocol shown in Figure S1B). Mice were then maintained in *ad libitum* feeding conditions for 3 days (days 28-30; no peanut butter).

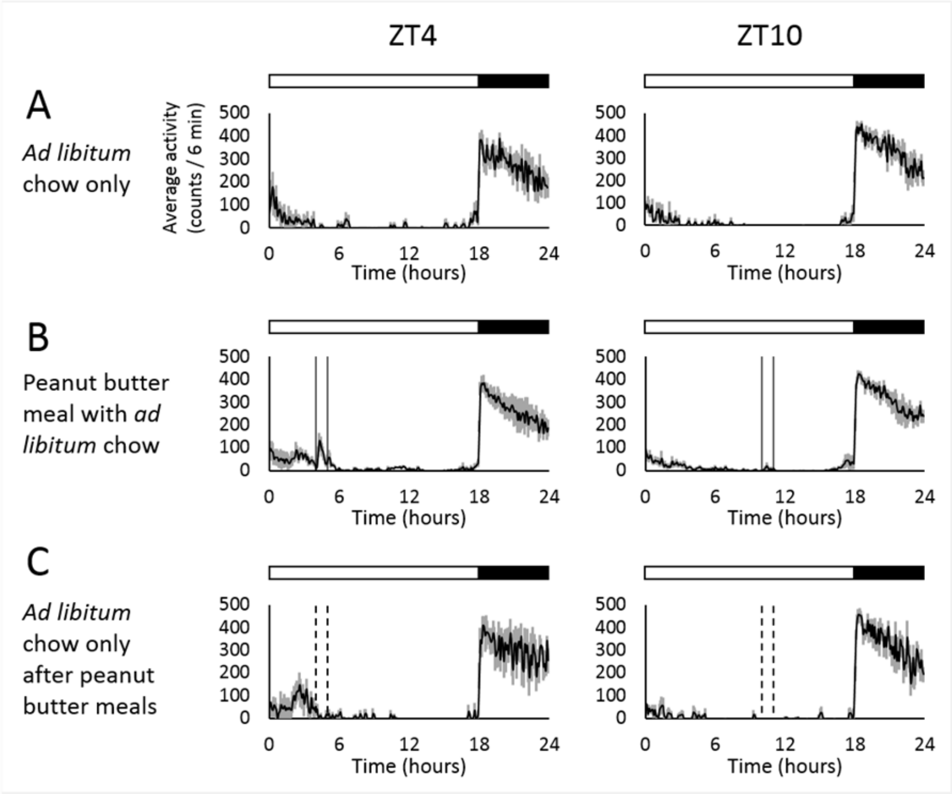

Figure S6. Group average activity profiles of the experiment presented in Figure 1 and S5). For graph specifications, see figure S3.

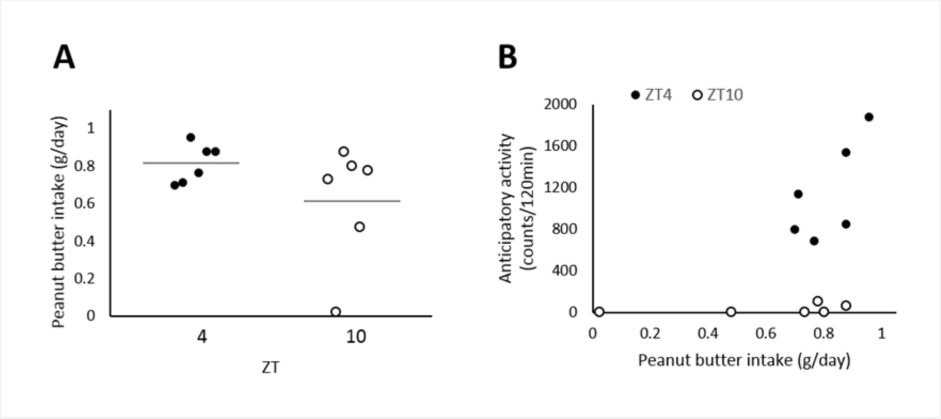

Figure S7. Peanut butter consumption is not correlated with the robustness of palatable meal anticipatory activity (from the experiment presented in Figure 1 and Figure S5). A: There is no difference in peanut butter consumption between the ZT4 and ZT10 groups (p=0.38 Mann-Whitney). B: There is no correlation between robustness of palatable meal anticipatory activity and peanut butter consumption (p=0.06, Spearman's correlation coefficient). See details in Figure S4, except that the last 9 days of peanut butter treatment were used to calculate peanut butter consumption, and all 14 days of peanut butter treatment were used to calculate anticipatory activity.

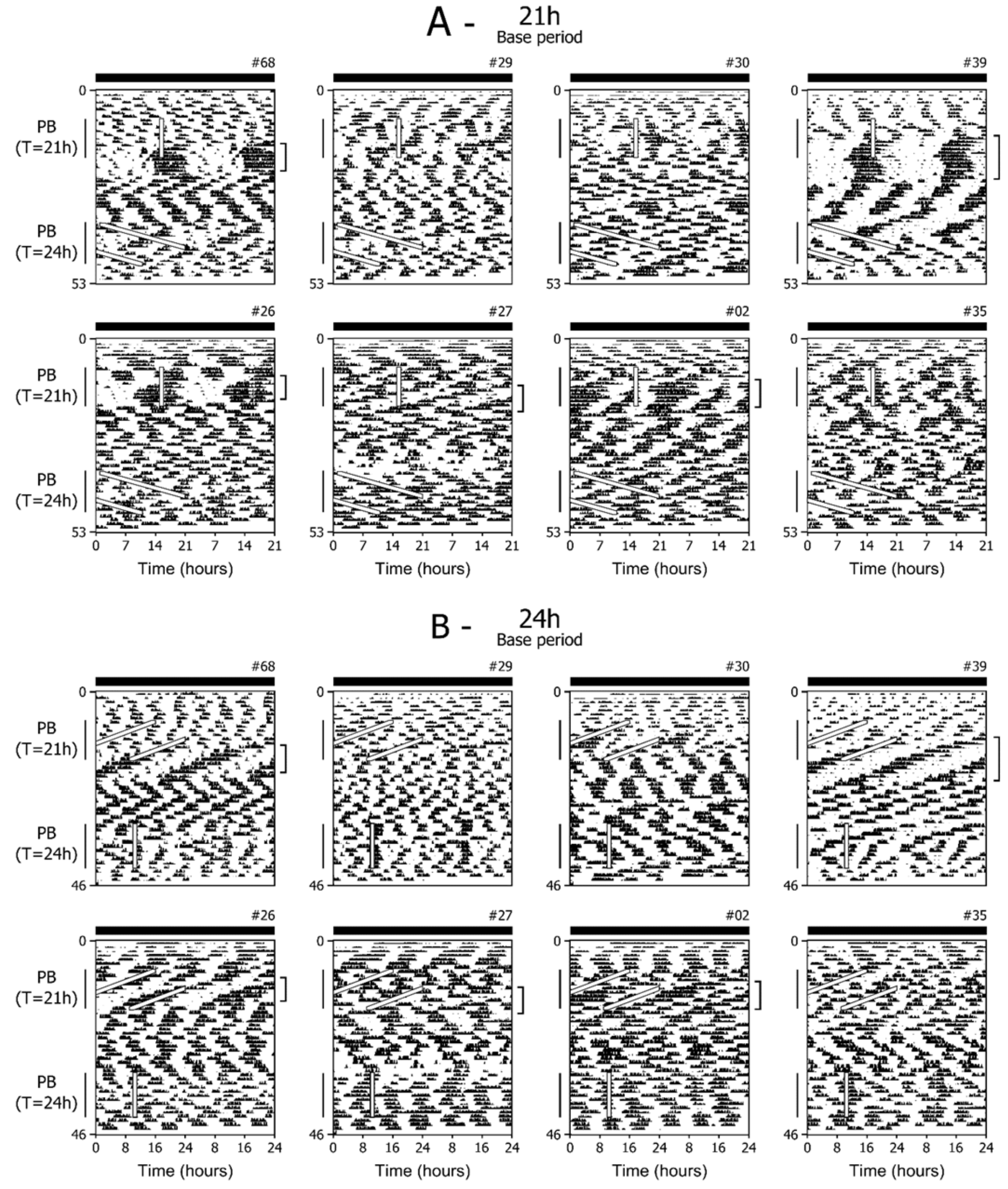
A - 21h Base period
#68
#29
#30
#39
#26
#27
#02
#35
PB (T=21h)
PB (T=24h)
0
53
0 7 14 21 7 14 21
Time (hours)
B - 24h Base period
0
46
0 8 16 24 8 16 24
Time (hours)

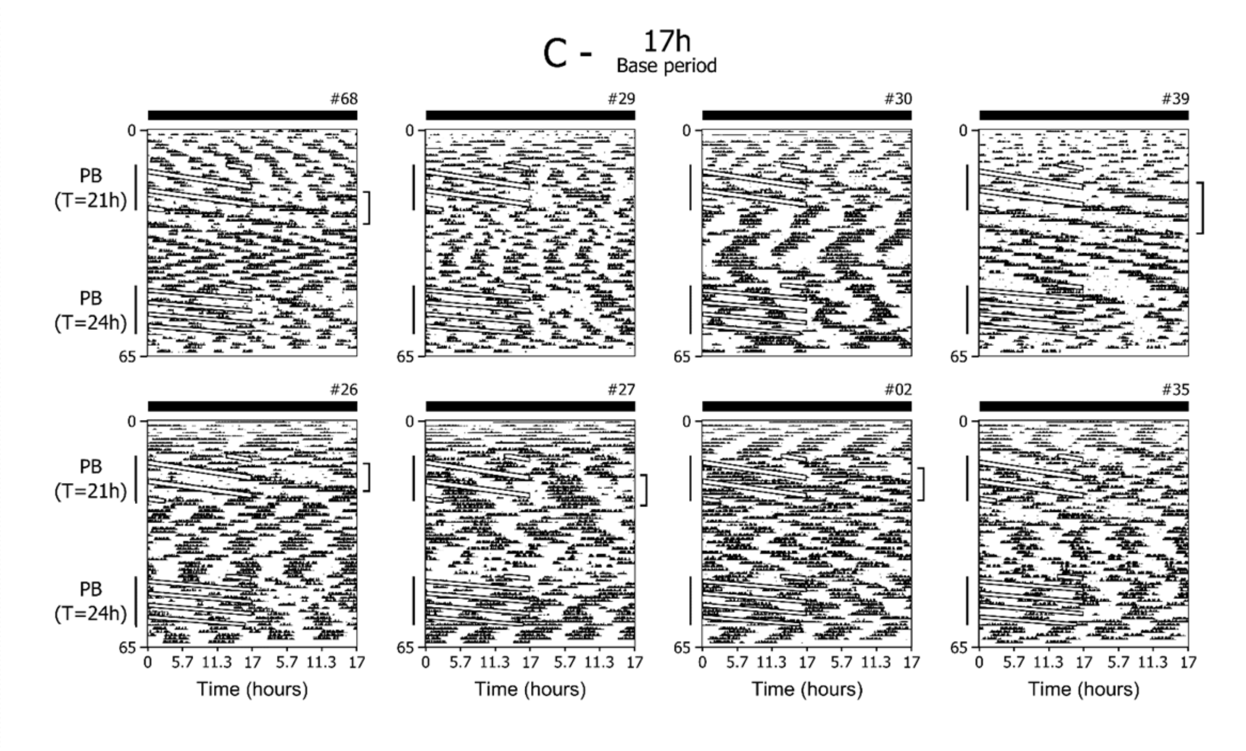

Figure S8. PICO in *Per1/2/3* triple mutant mice has a 21-h period and persists in constant conditions. Double-plotted actograms of wheel-running activity of *Per1/2/3* triple mutant mice kept in constant darkness with *ad libitum* chow throughout the experiment. Actograms are plotted with either a 21-h period (A), 24-h period (B), or 17-h period (C). Mice were fed peanut butter for 1 h each cycle on a 21-h cycle (T=21h) and then released into constant conditions (no peanut butter). Then mice were given peanut butter for 1 h each cycle on a 24-h cycle (T=24h). Brackets on the right of the graphs indicate the days used for period analysis (Table S1). Male: #68, 29, 30, 39. Female: #26, 27, 02, 35. Representative actograms were presented in Figure 2.

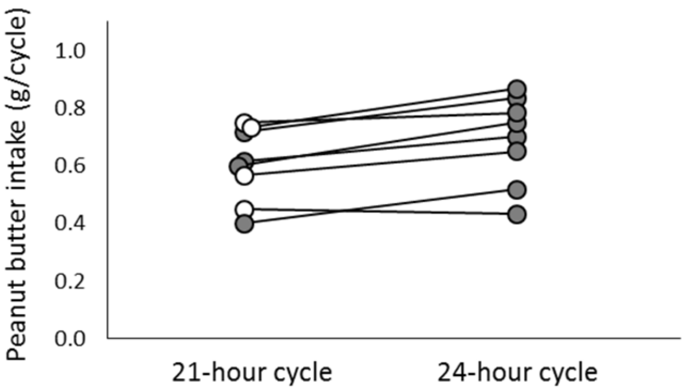

Figure S9. Peanut butter consumption of triple *Per1/2/3 triple* mutant mice. Average peanut butter consumption was calculated using the last 5 palatable meals in each treatment (21-hour cycle and 24-hour cycle). Each circle represents the average value for a single animal and the black lines connect the data for the same individual in the two treatments. Open circle: mouse developed consolidated activity rhythms. Closed circle: no consolidated activity rhythms developed.

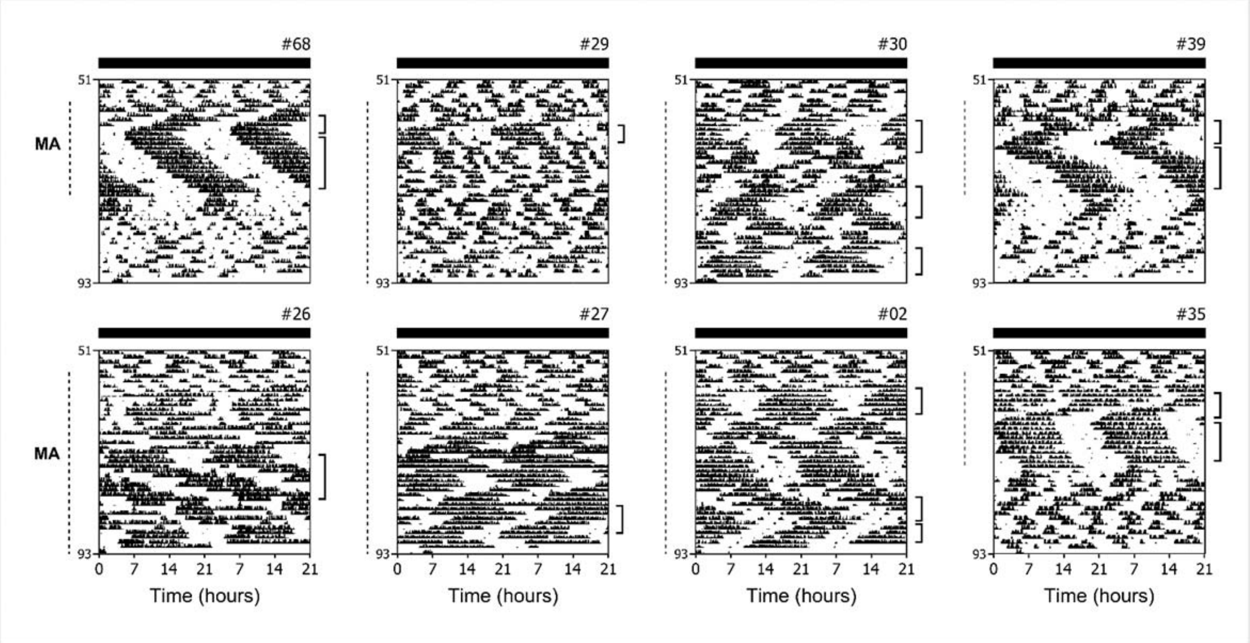

Figure S10. MASCO rhythm in *Per1/2/3* triple mutant mice. Double-plotted actograms (same mice are shown in Fig. S8) of the wheel-running activity of *Per1/2/3* mutant mice given methamphetamine (MA) in their drinking water (0.005%) in constant darkness. Actograms are plotted with a 21-h period (T21). Representative actograms were shown in Figure 3. Brackets on the right of the graphs indicate the days used for period analysis (Table S1).

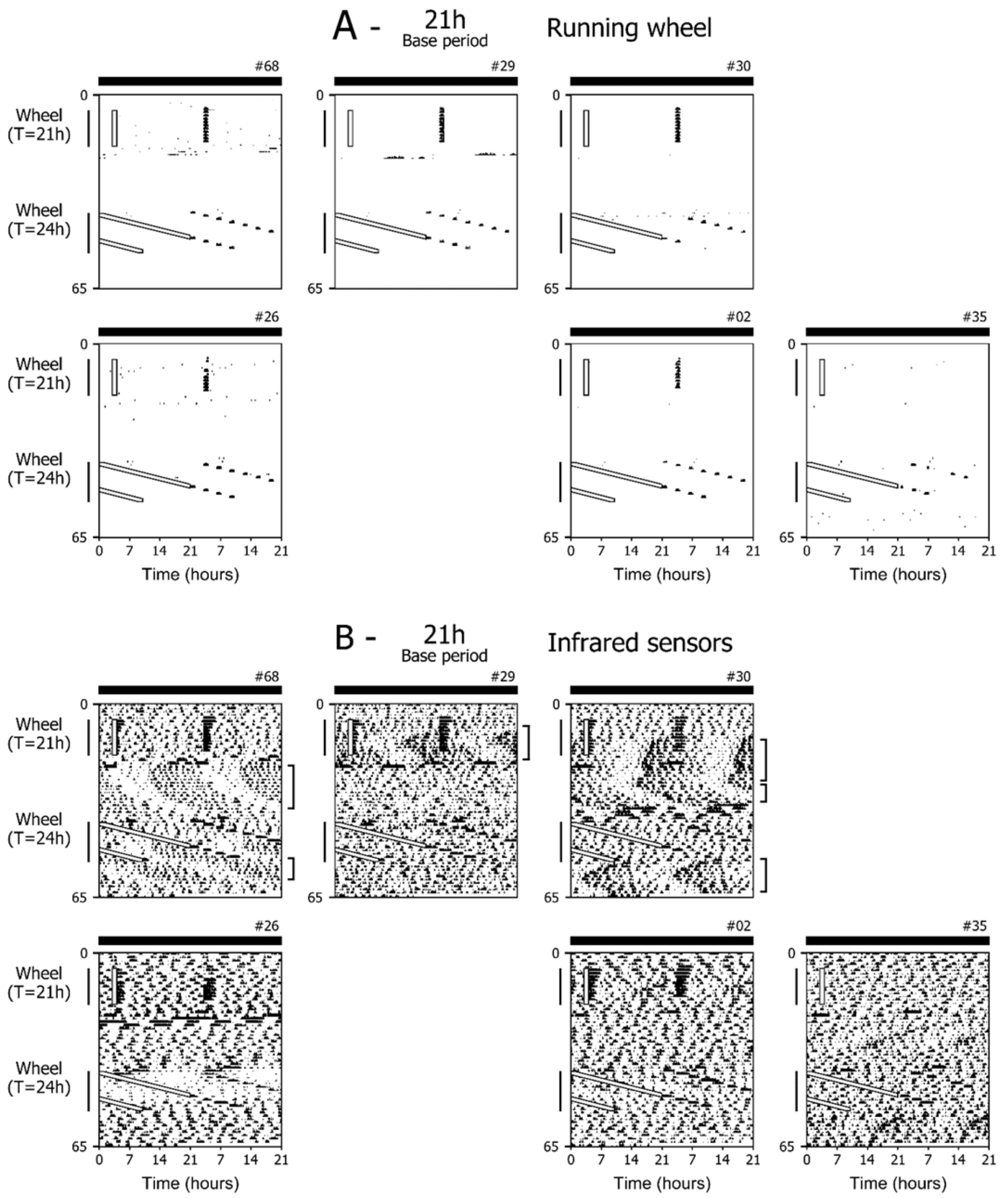

A - 21h Base period Running wheel
#68
#29
#30
#26
#02
#35
Wheel (T=21h)
Wheel (T=24h)
0
65
0 7 14 21 7 14 21
Time (hours)
B - 21h Base period Infrared sensors

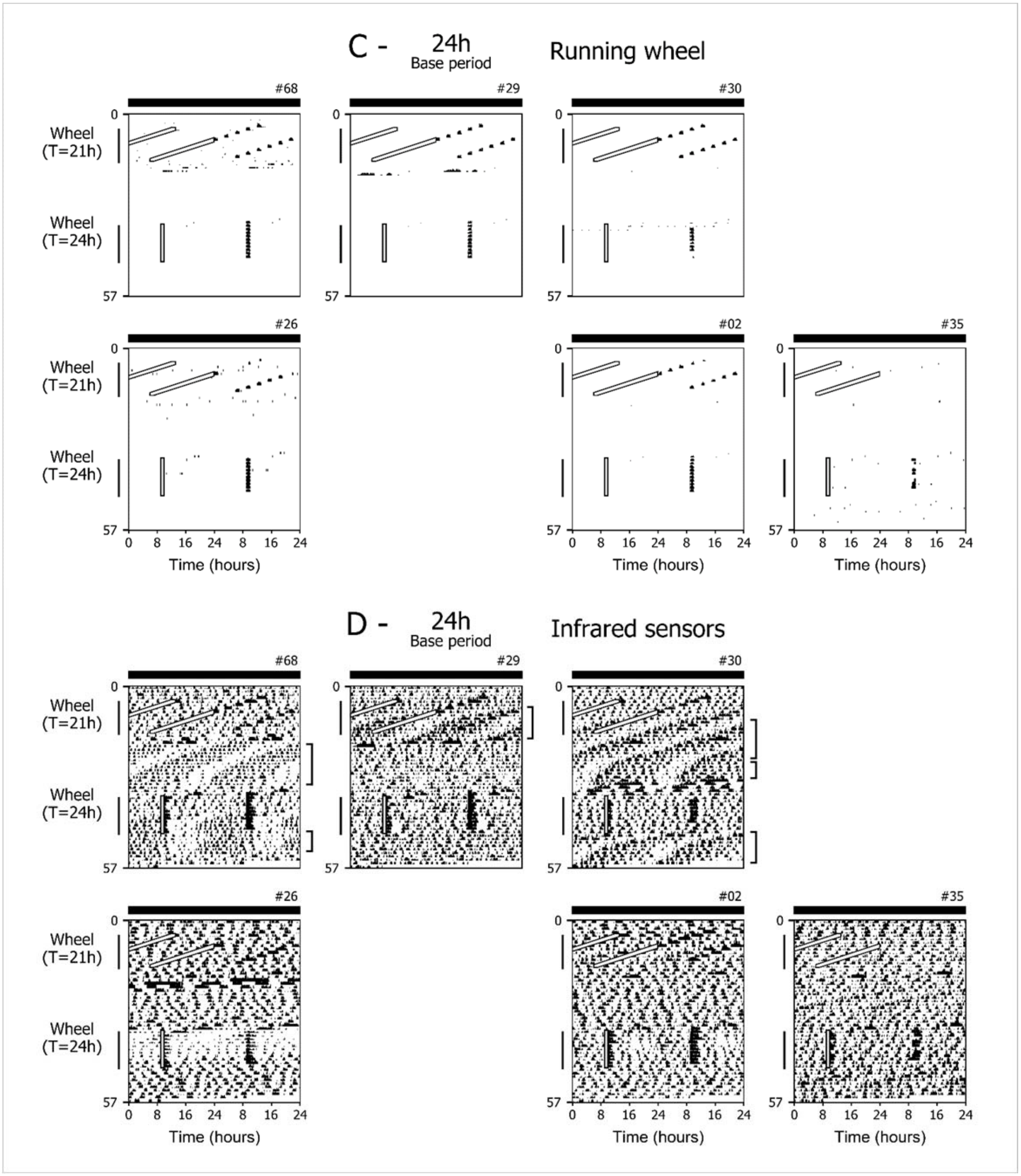

Figure S11. WICO rhythm is revealed in *Per1/2/3* triple mutant mice by periodic voluntary activity. Double-plotted actograms of wheel-running activity (A, C) and general activity (B, D, passive IR sensors) of *Per1/2/3* triple mutant mice in constant darkness with *ad libitum* chow. The wheel was automatically unlocked for 1 h each cycle on a 21-h interval (Wheel: T=21h; indicated by white box on left half of actograms) and then mice were released into constant conditions (continuously locked wheel). Then the wheel was unlocked for 1 h each cycle on a 24-h cycle (Wheel: T=24h) and then released into constant conditions. Actograms are plotted

with either a 21-h period (A, B) or 24-h period (C, D). Representative actograms were presented in Figure 4. Brackets on the right of the actograms indicate the days used for period analysis (Table S1). Mice #39 and #27 did not undergo this study because of a limited number of channels in the wheel locking system and a health-related concern.

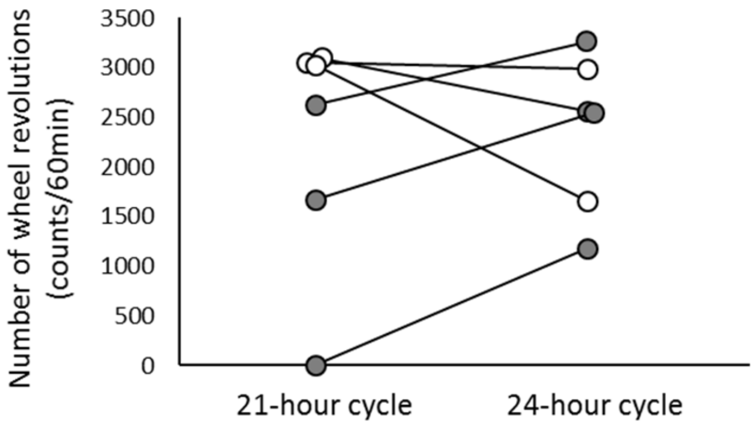

Figure S12. Wheel-running revolutions during 1 h of unlocked wheel. The number of wheel revolutions during 1 h of wheel unlocking in each mouse was obtained for each of the 11 cycles. Each circle represents the average wheel revolutions during the 11 cycles of wheel access for an individual mouse. Open circle: mouse exhibited activity consolidation (rhythmic). Closed circle: mouse did not show consolidated activity.
